# Negligible Impact of Perinatal Tulathromycin Metaphylaxis on the Developmental Dynamics of Fecal Microbiota and Their Accompanying Antimicrobial Resistome in Piglets

**DOI:** 10.3389/fmicb.2019.00726

**Published:** 2019-04-05

**Authors:** Mohamed M. Zeineldin, Ameer Megahed, Benjamin Blair, Brandi Burton, Brian Aldridge, James Lowe

**Affiliations:** ^1^Integrated Food Animal Management Systems, Department of Veterinary Clinical Medicine, College of Veterinary Medicine, University of Illinois at Urbana-Champaign, Champaign, IL, United States; ^2^Department of Animal Medicine, College of Veterinary Medicine, Benha University, Benha, Egypt

**Keywords:** antimicrobial, tulathromycin, microbiota, resistome, sequencing, piglets

## Abstract

While the antimicrobial resistance profiles of cultured pathogens have been characterized in swine, the fluctuations in antimicrobial resistance genes (ARGs) associated with the developing gastrointestinal microbiota have not been elucidated. The objective of this study was to assess the impact of perinatal tulathromycin (TUL) metaphylaxis on the developmental dynamics of fecal microbiota and their accompanying antimicrobial resistome in pre-weaned piglets. Sixteen litters were given one of two treatments [control group (CONT; saline 1cc IM) and TUL group (TUL; 2.5 mg/kg IM)] directly after birth. Deep fecal swabs were collected at day 0 (prior to treatment), and again at days 5 and 20 post treatment. Shotgun metagenomic sequencing was performed on the extracted DNA, and the fecal microbiota structure and abundance of ARGs were assessed. Collectively, the swine fecal microbiota and their accompanying ARGs were diverse and established soon after birth. Across all samples, a total of 127 ARGs related to 19 different classes of antibiotics were identified. The majority of identified ARGs were observed in both experimental groups and at all-time points. The magnitude and extent of differences in microbial composition and abundance of ARGs between the TUL and CONT groups were statistically insignificant. However, both fecal microbiota composition and ARGs abundance were changed significantly between different sampling days. In combination, these results indicate that the perinatal TUL metaphylaxis has no measurable benefits or detriment impacts on fecal microbiota structure and abundance of ARGs in pre-weaned piglets.

## Introduction

In swine production industry, antimicrobials are the most common prescribed drug primarily for treatment and prevention of diseases (Cromwell, 2002). The over use of existing antimicrobial results in perturbations of gut microbiota, promote the selection of antimicrobial-resistant microorganisms, and increase the abundance of various ARGs (Czaplewski et al., 2016; Hoelzer et al., 2017). Recently, there is widespread concern regarding the contribution of antimicrobial use in livestock to the development of antimicrobial resistance in people (Founou et al., 2016; Connelly et al., 2018). To overcome the resistance problem, the livestock production system must optimize the use of antimicrobial treatment (Maron et al., 2013). The key step in this optimization process is to understand the mechanism and extent by which antimicrobial intervention affects the resident microbiota, and their accompanying ARGs (Allen et al., 2014). Additionally, the ability to link the changes in the developmental dynamics of resident microbiota to their accompanying antimicrobial resistome is crucial in managing and preventing this global health threat. Most of the studies that evaluated the effect of antimicrobial interventions on the emergence of antibiotic resistant bacteria have frequently focused on phenotypic resistance in a single class of organism using culture methods (McEwen and Fedorka-Cray, 2002; Thanner et al., 2016). The advancements in high-throughput sequencing techniques, have improved our understanding about the gastrointestinal tract bacterial populations, and helped the researchers to quantitatively assess the dissemination of ARGs in different environments (Zhao et al., 2017).

Tulathromycin (TUL) is bacteriostatic macrolide act by inhibiting the biosynthesis of essential bacterial proteins and stimulates the disassociation of ribosomal peptidyl-tRNA during translocation process (Mazzei et al., 1993; Schokker et al., 2014). On the basis of its favorable antimicrobial characteristics, TUL is utilized therapeutically in neonatal piglets for control and prevention of infectious diseases at a single dosage of 2.5 mg/kg. BW (Pyörälä et al., 2014). Disruption of gut microbiota establishment and their accompanying antimicrobial resistome as a result of antimicrobial administration during this critical phase of production may produce important implications for swine health later in life (Kelly et al., 2017). Early-life TUL intervention in neonatal piglets exhibited limited effect on gastrointestinal microbial diversity and composition directly after administration but had a long-lasting impacts at day 176 after adiminstration (Schokker et al., 2014). Similarly, exploring the change of fecal microbiota of growing piglets in response to TUL administration revealed that the fecal microbiota structure exhibited a pronounced shift after single dose of treatment and had returned rapidly (within two weeks) to a distribution that closely resembled that observed on day 0 prior to treatment (Zeineldin et al., 2018). To fully understand the swine gastrointestinal microbial ecosystem during early life, it is important to understand the dynamics of gastrointestinal microbiota development and prevalence of ARGs in response to perinatal antimicrobial metaphylaxis. Consequently, the aim of this study was to investigate the short-term impact of perinatal TUL metaphylaxis on the developmental dynamics of fecal microbiota and their accompanying ARGs in neonatal piglets.

## Materials and Methods

### Ethics Statement, Animals and Samples Collection

The present study was conducted in a commercial swine farm in the Midwestern United States with consent from the facility owner. All procedures were carried out in agreement with principles and guidelines of the Institutional Animal Care and Use Committee of University of Illinois at Urbana-Champaign. The protocol was evaluated and approved by the Ethical Committee for Institutional Animal Use and Care of the University of Illinois at Urbana-Champaign. A total of 16 sows with their newborn piglets (220 piglets in total) were used in this study. Approximately five days before farrowing, the pregnant sows were transferred to the farrowing pens and kept there until the end of the experiment. Sows were given *ad libitum* water and fed a standard lactation diet via an automatic dry feeding system. No antimicrobials were administered to the sows before or after farrowing. Sows followed the normal farrowing procedures established by the farm and any piglet escaped this protocol was not enrolled in the study. Additionally, if more piglets were present than the dam milk glands, piglets were removed. All litters contained 12 to 14 piglets after this procedure. Directly after birth (< 6 h), litters were randomly assigned to one of two groups; CONT (*n* = 8 litters) and TUL group (*n* = 8 litters). In TUL group, a total of 108 piglets were treated with 2.5 mg TUL/kg IM (Draxxin^®^, Zoetis US, Chicago Heights, IL, United States). In CONT group, a total of 112 piglets were treated with saline 1cc IM. The piglet’s tails were docked, and 200 mg of Iron dextran was administered at three days of age. Males were surgically castrated at the same time according to the farm protocols. Daily physical examination was performed individually to evaluate the attitude and appetite of all piglets and their dams by farm staff. The piglets were individually identified with in litter. The weights of all piglets and mortality percent were recorded throughout the study. Individual deep fecal swabs (Pur-Wraps^®^, Puritan Medical Products, Guilford, Maine) were collected immediately prior treatment (day 0), and again on days 5 and 20 post treatment (Supplementary Figure S1). The fecal swabs were kept in dry ice-chilled boxes, transported to the laboratory on the same day and stored at -80°C until further processing.

### Fecal DNA Extraction and Shotgun Metagenomic Sequencing

Genomic DNA was extracted from subgroups of fecal swabs (4 piglets per sampling day in each group) and from negative control samples (sterile cotton swab and extraction kit reagent) using Power Fecal DNA Isolation kit (MO BIO Laboratories, Inc., Carlsbad, CA, United States) according to manufacturer’s standard protocol (Zeineldin et al., 2017a,b). The fecal swabs were randomly selected from the piglets that remained healthy throughout the sucking period. For each sample, total DNA concentration and integrity were evaluated using a Nanodrop^TM^ spectrophotometer (NanoDrop Technologies, Rockland, DE, United States) at wavelengths of 260 and 280 nm, and agarose gel electrophoresis (Bio-Rad Laboratories, Inc, Hercules, CA, United States). Extracted DNA was immediately stored at -20°C and then shipped on dry ice for sequencing at the W. M. Keck Center for Comparative and Functional Genomics (University of Illinois at Urbana-Champaign, Urbana, IL, United States).

DNA libraries were constructed using the Nextera DNA Flex Library Preparation Kit (Illumina, Inc., San Diego, CA, United States). Briefly, 100 ng of DNA were tagmented, cleaned up with magnetic beads and amplified for 5 cycles of PCR using Illumina Enhanced PCR Mix and Nextera FS dual indexed primers. Amplified DNAs were cleaned, and size selected for fragments 250 to 750 bp in length, using a double-sided bead purification procedure. The final libraries were quantitated using Qubit High-Sensitivity DNA (Life Technologies, Grand Island, NY, United States) and the average size was determined on the AATI Fragment Analyzer (Advanced Analytics, Ames, IA, United States). Libraries were pooled evenly, and the pool was cleaned using a 1:1 ratio with AxyPrep Mag PCR Cleanup beads (Axygen, Inc., Union City, CA, United States), then evaluated again on AATI Fragment Analyzer (Advanced Analytics, Ames, IA, United States). The final pool was diluted to 5 nM concentration and further quantitated by qPCR (Bio-Rad Laboratories, Inc., CA, United States). The pool was then denatured and spiked with 4% non-indexed PhiX control library and loaded onto the MiSeq V3 flowcell at a concentration of 10 pM for cluster formation and sequencing. Finally, DNA libraries were sequenced from both ends of the molecules to a total read length of 250 nt from each end following manufacturer’s guidelines (Illumina, Inc., San Diego, CA, United States).

### Sequence Data Processing and Microbial Community Analysis

Raw sequence data files were de-multiplexed and converted to fastq files using Casava v.1.8.2 (Illumina, Inc. San Diego, CA, United States). Sequence reads quality were assessed using FastQC software (Andrews, 2010). Adaptor sequence and low-quality reads with Phred score <30 were trimmed from the raw sequence data using Trimmomatic software (Bolger et al., 2014). The trimmed sequence files were then uploaded to the Metagenome Rapid Annotation Using Subsystems Technology (MG-RAST) webserver to determine the taxonomic composition of fecal microbiota at the phylum, genus, and species levels, and to predict the metabolic functional gene profiles (Glass et al., 2010). The MG-RAST webserver utilizes a high-performance data-mining algorithm along with curated genome databases that rapidly disambiguates millions of short reads of a metagenomics sequence into discrete microorganisms engendering the identified sequences. In MG-RAST, sequence reads were subjected to additional quality control filtering, including dereplication (removal of sequences produced by sequencing artifacts), removal of host-specific species sequences, length filtering (removal of sequences with a length > 2 standard deviations from the mean), and ambiguous base filtering (removal of sequences with > 5 ambiguous base pairs). Normalization was performed using a log_2_-based transformation [log_2_ (x + 1)], followed by standardization within each sample and linear scaling across all samples (Gaeta et al., 2017). We used a nonredundant multisource protein annotation database (M5NR) as annotation source for microbial classification. Microbiota abundance was analyzed using a best-hit classification approach with a maximum e value of 1 × 10^-5^, a minimum identity cutoff of 60%, and a minimum alignment length cutoff of 15. We used SEED subsystem as the annotation source for predicted metabolic functional gene profiles. To be publicly available, the sequence reads were deposited in MG-RAST webserver under the following accession numbers: from mgm4779141.3 to mgm4779164.3.

Fecal microbiota alpha diversity indices were calculated within PAST version 3.13 using Chao 1, Shannon, Simpson and Evenness indices. Beta diversity was computed using principal component analysis (PCA) based on non-phylogenetic Bray–Curtis distance metrics implemented in MicrobiomeAnalyst (Dhariwal et al., 2017). The difference in overall microbial composition between the CONT and TUL groups was determined using non-parametric multivariate analysis of variance (PERMANOVA) with 9999 permutations and Bonferroni corrected *P* values in PAST version 3.13. The difference in fecal microbiota relative abundance and alpha diversity metrics between the two groups (CONT and TUL) at each time point (Day 0, 5, and 20) were analyzed using Mann–Whitney pairwise comparison test with sequential Bonferroni significance in PAST version 3.13. Significance difference was stated at *P* < 0.05. To further quantify the overall microbial composition similarities between the two groups at each time point, the relative abundance of fecal microbiota at genus level were assessed using the linear discriminant analysis effect size (LEfSe) pipeline using Galaxy^1^ (Segata et al., 2011). The difference in overall predictive function gene profiles between the CONT and TUL groups were compared using STAMP (Statistical Analysis of Metagenomic Profiles) software (Parks et al., 2014). For two-groups analysis, two-sided Welch’s *t*-test and Benjamini–Hochberg FDR correction were used, while for multiple-groups analysis, ANOVA with the Tukey-Kramer test and Benjamini–Hochberg correction were chosen. Differences were stated significant at *P* < 0.05. PCA and heatmap diagram were also performed using STAMP software.

### Antimicrobial Resistance Genes Identification

To assess and quantify the relative abundance of the ARGs in our data, we used SRST2 pipeline (Inouye et al., 2014). The SRST2 pipeline was used to map the raw sequence reads and cluster the similar sequences against a database of preference, using CD-hit with an identity threshold of 80% (Clausen et al., 2016). For ARGs identification, we used antibiotic resistance gene database (ARG-ANNOT) that incorporated all sequences of known antibiotics resistance genes (Lopez-Rojas et al., 2013). Antimicrobial resistance genes alpha diversity metrics were computed using the Shannon index, Simpson’s index, Chao1 richness estimate and Pielou’s evenness index. The difference in the relative abundance and diversity of ARGs between the CONT and TUL groups at the different sampling days were analyzed using Mann–Whitney pairwise comparison test with sequential Bonferroni significance in PAST version 3.13. Additionally, two-sided Welch’s *t*-test and Benjamini–Hochberg FDR correction were used to compare the overall difference in ARGs abundance between the CONT and TUL groups using STAMP software (Parks et al., 2014). Differences were considered significant at *P* < 0.05. PCA and heatmap diagram were also performed using STAMP software. The overall difference in ARGs abundance between the CONT and TUL groups was determined using PERMANOVA with 9999 permutations and Bonferroni corrected *P* values in PAST version 3.13.

## Results

### Impact of TUL Metaphylaxis on the Body Weight Gain and Overall Mortality Percent

There was no significant change in the average daily weight gain between day 0 and day 20 in the TUL group compared to CONT group (means ± SE; 4.61 ± 0.18 vs. 4.54 ± 0.26, Supplementary Figure S2A). The TUL-treated piglets showed also non-significant changes in the overall mortality rate (day 0 to 20) compared to the CONT (means ± SE; 0.028 ± 0.005 vs. 0.021 ± 0.009, Supplementary Figure S2B). Our results showed that the early-life TUL administration has no advantage in increasing the average daily weight gain in the neonatal piglets or reducing the piglet’s mortality during the neonatal periods.

### Shotgun Metagenomic Sequencing Summary

Across all fecal samples, shotgun metagenomic sequencing generated a total of 19,236,952 raw sequence reads (mean number of sequences per sample: 400,742.88; median: 394,675; range: 358,524–464,985). The average Phred quality score of raw sequence reads across all samples was 33.7 and only 1.01% of all reads were removed due to low quality. Using the criterion of MG-RAST taxonomic classification, 3,833,882 taxonomic hits were identified among all samples, all of which were taxonomically assigned according to RefSeq classification. Collectively, a total of 2,010,187 and 1,829,585 hits were identified in the CONT and TUL piglets, respectively.

### Taxonomical Classification of the Fecal Microbiota

**FIGURE 1:**
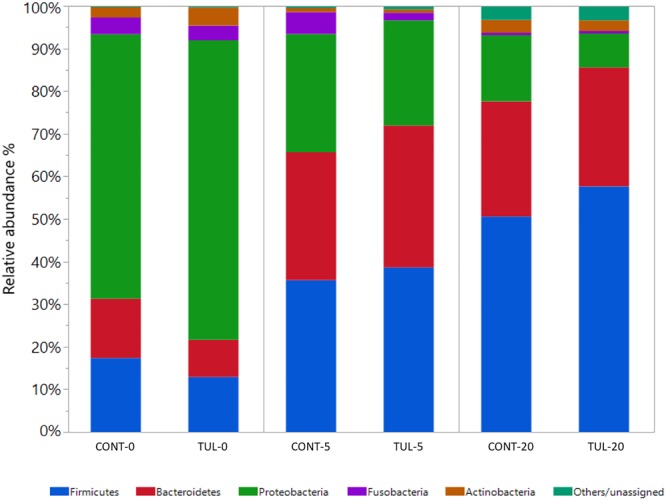
Taxonomic classification of shotgun metagenomic sequences at the phylum level for the control (CONT) and tulathromycin (TUL) treated piglets at each sampling time days (0, 5, and 20). Only those bacterial phyla that averaged more than 1% of the relative abundance across all samples are displayed.

Across all samples, 29 different bacterial phyla, 586 genera, and 1468 species were detected using MG-RAST webserver. Collectively, the fecal microbiota composition at both phylum and genus level in the CONT and TUL piglets varied greatly according to the age. At the phylum level, *Proteobacteira* was the most predominant phylum at day 0, representing 62 and 70% of all bacterial populations in CONT and TUL- treated piglets, respectively. While at day 20, *Firmicutes* was the most abundant phylum, representing 52 % and 60 % of all bacterial populations in the CONT and TUL-treated piglets, respectively. Distribution of the most abundant bacterial phyla in both CONT and TUL groups at different sampling days are depicted in (Figure 1). When selectively comparing changes between the CONT and TUL-treated piglets, there was no significant change in bacterial phyla that averaged more than 1% of the relative abundance.

**FIGURE 2:**
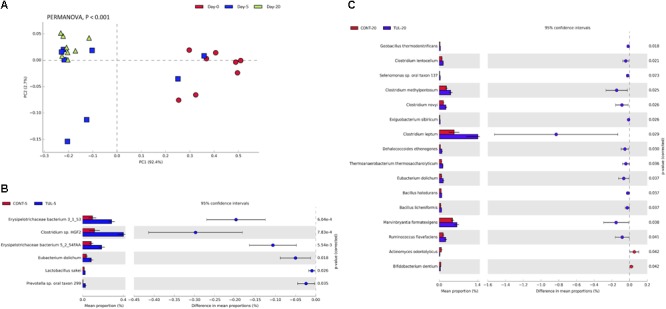
**(A)** Principal component analysis (PCA) for the microbial composition at the species level across all samples at different sampling days (0, 5, and 20). The percent variation explained by each component is indicated on the axes. Significance between groups was analyzed using PERMANOVA with 9999 permutations and Bonferroni corrected *P-*values. **(B)** Bacterial species that showed significant difference when compared the control (CONT) and tulathromycin (TUL) piglets at days 5. **(C)** Bacterial species that showed significant difference when compared CONT and TUL-treated piglets at days 20.

At the genus level, the predominant bacterial genera that averaged more than 1% across all samples at the baseline (day 0) was comprised of common fecal microbial genera including *Escherichia* (50.72%), *Bacteroides* (7.73%), *Clostridium* (7.03%), *Shigella* (5.61%), *Streptococcus* (2.18%), *Fusobacterium* (1.74%), *Salmonella* (1.31%), and *Lactobacillus* (1.01%). Distribution of the most abundant bacterial genera in both CONT and TUL groups at different sampling days are depicted in (Supplementary Figure S3). Even though there were no significant changes detected in bacterial genera that averaged more than 1% between the two groups, in-depth analysis at genera-level suggested that TUL treatment was associated with minor changes in the fecal microbiota of these young piglets. At the species level, the most predominant 100 microbial species across all samples are depicted in (Supplementary Table S1). Collectively, the microbial composition at species level in the CONT and TUL piglets varied greatly according to the age (Figure 2A). Additionally, the relative abundance of some bacterial species showed significant difference when compared to the CONT and TUL-treated piglets at days 5 and 20 (Figure 2B,C).

**FIGURE 3:**
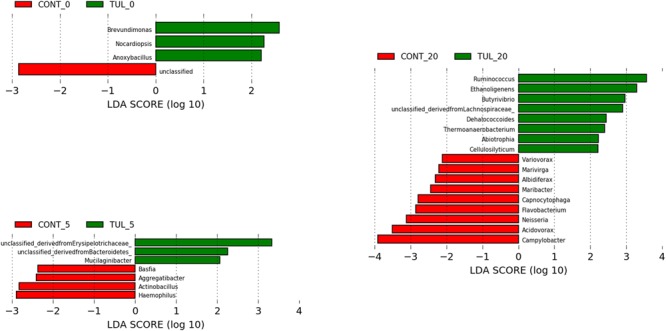
Identification of indicator bacterial taxa associated with statistically significant differential abundance between the control (CONT) and tulathromycin (TUL) piglets at the different sampling days (0, 5, and 20). The top OTUs with the highest LDA score log10 ≥ 2.0 that discriminate between the CONT and TUL treated piglets at each time point are depicted.

Based on LEfSe algorithm, the changes in the fecal microbiota structure caused by perinatal TUL intervention are limited to a particular group of microbial taxa (Figure 3). Compared to the CONT group, 3, 3 and 8 OTUs were identified as indicator taxa in the TUL-treated piglets at days 0, 5, and 20, respectively (Figure 3). At day 5, the TUL-treated piglets exhibited a high contribution of *Erysipelotrichaceae, Bacteroidetes*, and *Mucilaginibacter* taxa. While at day 20, *Ruminococcus, Ethanoligenens, Butyrivibrio, Lachnospiraceae, Dehalococcoides, Thermoanaerobacterium, Abiotrophia*, and *Cellulosilyticum* taxa were enriched in the TUL piglets.

**FIGURE 4:**
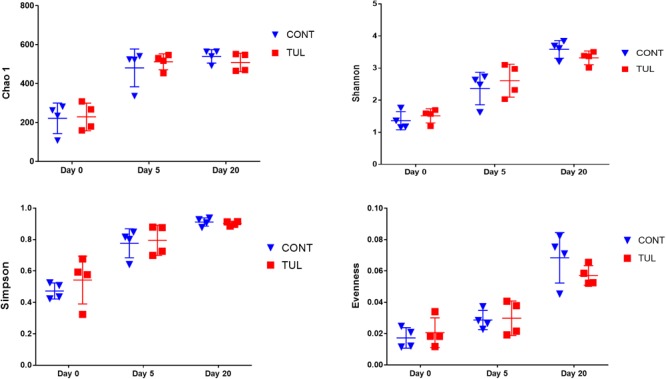
The difference in bacterial diversity indices (Chao 1, Shannon, Simpson and Evenness) measures between the control (CONT) and tulathromycin (TUL) groups at different sampling days (0, 5, and 20). The individual data points, which represent bacterial diversity for each piglet, are depicted. Error bars represent the standard errors.

**FIGURE 5:**
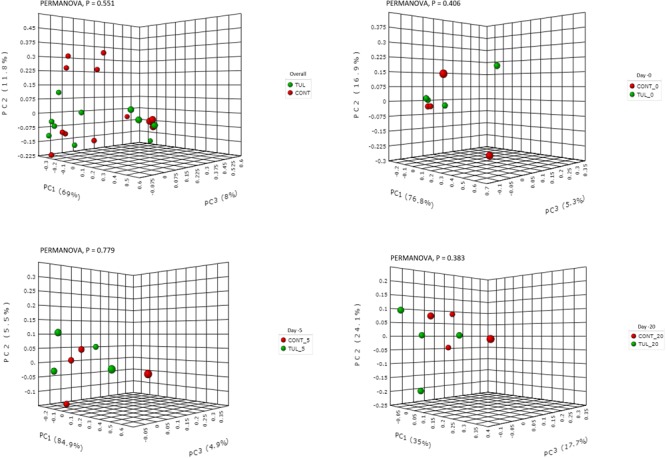
Principal component analysis (PCA) based on non-phylogenetic Bray–Curtis distance metrics for control (CONT) and tulathromycin (TUL) piglets at different sampling days (0, 5 and 20). The percent variation explained by each principal component is indicated on the axes. Significance between the two groups was analyzed using PERMANOVA with 9999 permutations and Bonferroni corrected *P-*values.

We next investigated the effects of early life TUL metaphylaxis on the fecal microbiota diversity. Alpha diversity metrics showed non-significant changes between the CONT and TUL groups (Figure 4). However, the metagenomic analysis in both experimental groups revealed that the microbial diversity and richness indices were increased with the age (Figure 4). Beta diversity analysis also showed that the TUL-induced changes in the microbial community composition were not sufficient to cluster the microbial populations at days 0, 5, and 20 as shown by PCA of Bray–Curtis distance (Figure 5).

### Effect of TUL Metaphylaxis on Microbial Functional Profiles

**FIGURE 6:**
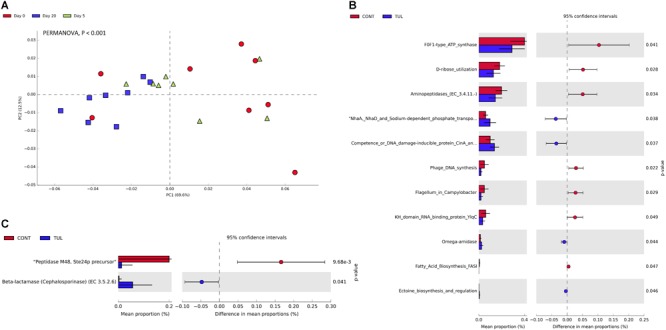
**(A)** Principal component analysis (PCA) for the predicted functional profiles at level 3 across all samples at different sampling days (0, 5, and 20). The percent variation explained by each component is indicated on the axes. Significance between groups was analyzed using PERMANOVA with 9999 permutations and Bonferroni corrected *P-*values. **(B)** Extended bar plot showed the statistically significant difference in functional gene features in the tulathromycin (TUL) piglets compared to the control (CONT) group. *P*-value < 0.05 was considered significant. **(C)** Extended bar plot showed the statistically significant difference in the resistance to antibiotics functional genes in the tulathromycin (TUL) piglets compared to the control (CONT) group. *P*-value < 0.05 was considered significant.

The relative abundance of the microbial functional profiles at level 2 KEGG pathway is depicted in (Supplementary Figure S4A). There was no significant difference in the overall metabolic functional capability at level 2 pathway between the CONT and TUL groups (Supplementary Figures S4B,C). However, the overall predicted functional profiles in both CONT and TUL were varied greatly according to the age (Figure 6A). Furthermore, the extended bar plot of the functional potential at level 3 KEGG pathways revealed significant difference in the relative abundance of some metabolic and antibiotic resistance functional genes between the CONT and TUL-treated piglets (Figure 6B,C).

### Effect of TUL Metaphylaxis on Antimicrobial Resistance Genes

**FIGURE 7:**
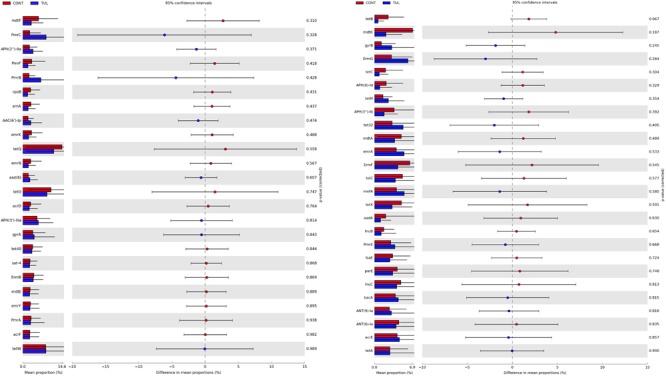
Extended bar plot showed the 50-predominant antimicrobial resistance genes, between the control (CONT) and tulathromycin (TUL) groups. *P*-value < 0.05 was considered significant.

Across all samples, a total of 127 ARGs related to 19 different classes of antibiotics were identified. The detected ARGs confer resistance to lipopeptide, aminocoumarin, tetracycline, fluoroquinolone, beta-lactam, aminoglycoside, streptogramin, macrolide, lincosamide, lipopeptide, rifamycin, phenicol, peptide, glycopeptide, nucleoside, sulfonamide, fluoroquinolones, coumarin, rifampin, and diaminopyrimidine antibiotics. A heatmap of identified ARGs relative abundance in the fecal microbiota at class level in both CONT and TUL groups was depicted in (Supplementary Figure S5). The identified ARGs were observed in both CONT and TUL groups and at all-time points. The highest level of ARGs across all samples were associated with *tetQ* (10.22%), *tetO* (7.21%), and *tetW* (6.24%), *PmrC* (4.65%), and *APH(3′)-IIIa* (3.77%). The magnitude and extent of differences in the 50-predominant ARGs, between the CONT and TUL groups were statistically insignificant (Figure 7).

**FIGURE 8:**
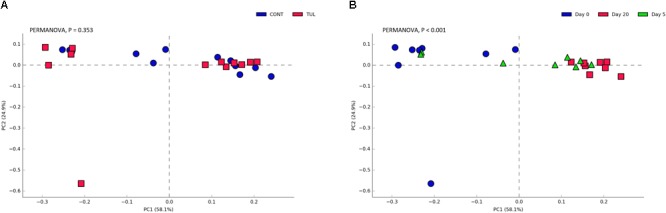
**(A)** Principal component analysis (PCA) based on non-phylogenetic Bray–Curtis distance metrics for the overall fecal antimicrobial resistance genes between the control (CONT) and tulathromycin (TUL) piglets. **(B)** Principal component analysis (PCA) for the overall fecal antimicrobial resistance genes across all samples at different sampling days (0, 5, and 20). The percent variation explained by each component is indicated on the axes. Significance between the two groups was analyzed using PERMANOVA with 9999 permutations and Bonferroni corrected *P-*values.

To gain further insight, we calculated several alpha-diversity indices for ARGs in both CONT and TUL groups. Alpha diversity analysis showed non-significant changes in the Chao1, Shannon, Simpson and Pielou’s evenness indices between the CONT and TUL groups (Supplementary Figure S6). However, the metagenomic analysis revealed that the ARGs diversity and richness indices were increased with age. PCA also showed that the overall fecal ARGs did not differ significantly between the TUL and CONT groups (PERMANOVA, *P* = 0.353; Figure 8A). However, the ARGs abundance across all samples varied greatly according to the age (PERMANOVA, *P* < 0.001; Figure 8B).

## Discussion

In this study, we used shotgun metagenomic sequencing to assess the developmental dynamics of fecal microbiota and their accompanying antimicrobial resistome in the newborn piglets in response to TUL metaphylaxis soon after birth. This study was performed in a commercial swine farm to improve the practical relevance of our results. The findings of this study revealed that single dose of TUL prophylaxis immediately after birth had no advantage in reducing the mortality and/or increasing the average daily weight gain in the neonatal piglets. The early-life microbial composition soon after birth was predominantly comprised of *Escherichia, Bacteroides, Clostridium, Shigella, Fusobacterium*, and *Streptococcus*. These taxa create an anaerobic environment that play an important role in establishing the other health beneficial strict anaerobes (Pantoja-Feliciano et al., 2013). The piglets fecal microbiota composition observed in this study soon after birth was similar to that published by (Rodríguez et al., 2015; Slifierz et al., 2015; Kubasova et al., 2017; Maradiaga et al., 2018). In 20-day-old piglets, *Clostridium, Bacteroides, Escherichia, Lactobacillus*, and *Prevotella* were the most abundant microbiota member, and were similar to the previous reports (Slifierz et al., 2015; Kubasova et al., 2017). While our study revealed that the age is the most significant contributor in the fecal microbiota development, understanding the early colonization pattern of gut microbiota will open the door to new perspectives related to the impacts of early life antimicrobials administration on the health of neonates in the swine management systems.

While, there have been contradictory reports regarding the impact of antimicrobial interventions on fecal microbiota, our results are broadly consistent with a previous study that assessed the effects of TUL intervention on fecal microbiota and their accompanying ARGs in commercial feedlot calves (Doster et al., 2018). Doster and his colleagues reported that the fecal microbiota structure and ARGs relative abundance were not significantly different between the control and TUL-treated cattle using shotgun metagenomic sequencing. Similarly, our results revealed that the single dose of TUL metaphylaxis in the neonatal piglets has no measurable benefits or detriment impacts on either the overall fecal microbial community or emergence of ARGs.

In this study, the use of TUL metaphylaxis was associated with non-significant changes in microbial diversity compared to the CONT piglets. Similar to our findings, antibiotic administration in cattle showed non-significant changes the fecal microbiota composition and diversity in the lower gastrointestinal tract (Thomas et al., 2017). This might be due to the rapid absorption and distribution of TUL from the injection site to the target tissues particularly the respiratory tract, with exceptionally long elimination half-life in the lung tissue (6 days in pigs) (Benchaoui et al., 2004). Moreover, TUL excretion is somewhat slow (about 70% within 23 days), with the excreted route being divided between urine (40%) and feces (32%). The modest changes in the fecal microbiota composition following early life TUL metaphylaxis are likely to reflect a combination between the resident microbiota resistance mechanism and the relatively weak gastrointestinal selective pressure of single-dose of TUL (Choo et al., 2018).

Similar to the highly diverse and developed fecal microbiota composition, the overall predicted functional profiles in both CONT and TUL-treated piglets varied greatly according to the age. The age variability in functional profiles has also been previously detected in RNA and DNA -based metagenomic analysis (Phillips et al., 2004; Qin et al., 2010). While these are only statistical presumptions in functional features of the taxonomically assigned microbial population in our study, similar changes have been declared after antimicrobial treatment in human (Pérez-Cobas et al., 2013). Further investigations into the functional profiles either by direct metabolites measurement or by transcriptome analysis will be an essential next step to better understand the effect of the early life antimicrobial interventions on gut microbiota function in swine.

One important consequence of overuse of antimicrobials in livestock production is the spread of ARGs, which is a serious public health issue (MacKie et al., 2006). Recently, the use of functional metagenomic provides a potential resource for detecting the existence of ARGs in gastrointestinal microbial community (Thomas et al., 2017). In this study, the identified ARGs were observed in both CONT and TUL-treated piglets across all-time points. Similarly, previous studies have reported that the newborn infants harbor ARGs that potentially acquired from their mothers (Yassour et al., 2016). Some ARGs were also detected in the absence of antimicrobial exposure in both human (Tsukayama et al., 2018) and cattle (Chambers et al., 2015). Interestingly, the magnitude and extent of differences in the proportion of macrolide resistance genes sequence between the TUL and CONT groups were statistically insignificant (*P* > 0.10; Figure 8). Similarly, macrolide treatment did not result in a significant increase in the macrolide resistance genes (*erm(A), erm(B), erm(C), erm(F), mef(A/E)*, and *msrA* in people (Choo et al., 2018). In combination, there was no measurable effect of TUL treatment on ARGs in this group of piglets. Since we used only single dose of TUL and the total study duration in this study was only 20 days, the ARGs profile we determined here may not be representation of longer-term effect of such antimicrobial metaphylaxis.

While the results of this study could open a new avenue in understanding the impact of antimicrobial administration on the early-life developmental dynamics of fecal microbiota and resistome in piglets, our study had number of experimental limitations that should be considered. Our analysis focused only on the fecal microbiota and their accompanying ARGs. Whereas the impact of antimicrobial treatment on microbiota within other gastrointestinal regions is likely to be consistent with the results reported here, changes in composition and ARGs characteristics in other commensal populations in different biogeographic locations should be considered. Our analysis also focused on the short-term impact of TUL administration on fecal microbiota (first 20 days of life). It would have been interesting to continue to sample the piglets for a longer period after weaning to define how these minor changes impact the future health and productivity of growing piglets. Finally, the major limitation in this study was the low sequence reads and sequencing depth per sample compared to other metagenomic study (Ferguson et al., 2013). Despite these experimental limitations, our study results provide preliminary insight into an area of investigation that could be of great relevance to swine gut health.

## Conclusion

This study demonstrated that TUL metaphylaxis at birth had relatively minor effects on the developmental dynamics of gut microbiota and their accompanying antimicrobial resistome in suckling piglets. This study suggests that a single dose of metaphylactic TUL treatment may be employed at birth without incurring drastic changes to the fecal microbiota and their accompanying ARGs in swine. However, further long-term studies across larger populations should be conducted to determine the beneficial and/or the detrimental effects of early life antimicrobials prophylaxis on gut microbial community structure and ARGs in pigs. Understanding when and how the gut microbiota responds to the antimicrobial administration will open the door to new perspectives on the utility of early life antimicrobial to healthy neonates in our livestock management systems.

## Ethics Statement

The present study was conducted in a commercial swine farm in the Midwestern United States with consent from the facility owner. All procedures were carried out in agreement with principles and guidelines of the Institutional Animal Care and Use Committee of University of Illinois at Urbana-Champaign. The protocol was evaluated and approved by the Ethical Committee for Institutional Animal Use and Care of the University of Illinois at Urbana-Champaign.

## Author Contributions

JL and BA designed the experiments. BBl, BBu, AM, and MZ conducted the experiments. MZ, BBl, and BBu performed the laboratory analyses. MZ and AM conducted the data analysis and wrote the manuscript. JL and BA edited the manuscript. All authors approved the manuscript submission.

## Conflict of Interest Statement

The authors declare that the research was conducted in the absence of any commercial or financial relationships that could be construed as a potential conflict of interest.

## Footnotes

1 https://huttenhower.sph.harvard.edu/galaxy/

## Supplementary Material

FIGURE S1 The detailed experimental design.

FIGURE S2 **(A)** Bar graph illustrating the body weight (kg) at day 0 and day 20, and the average weight gain from day 0 to day 20 of age. **(B)** Bar graph illustrating the Mortality percent of piglets from day 0 to day 5 (day 0–5), from day 5 to day 10 (day 5–10), from day 10 to day 15 (day 10–15), and from day 15 to day 20 (day 15–20). The piglets were treated with a single dose of TUL at day 0 soon after birth (TUL, *N* = 108), or treated with saline (CONT, *N* = 113). There was no significant change in the average daily weight gain and overall mortality ratio (*P* > 0.05).

FIGURE S3 Taxonomic classification of shotgun metagenomic sequences at the genus level for the control (CONT) and tulathromycin (TUL) treated piglets at each sampling time days (0, 5, and 20). Only those bacterial genera that averaged more than 1% of the relative abundance across all samples are displayed.

FIGURE S4 Inferred predictive functional features of piglet’s fecal microbiota. **(A)** Heatmap cluster analysis of metagenomic functional capability at level 2 KEGG pathway based on differentially abundant functional features between the control (CONT) and tulathromycin (TUL) groups, and at different sampling days (0, 5, and 20). The yellow/blue color of the X axis of the heat map represent the degree of similarities and cluster between the related class of assessed parameters. **(B)** The relative abundance of the functional profiles at level 2 in the TUL treated piglets compared to the CONT group (*P*-value > 0.05). **(C)** Principal component analysis (PCA) based on non-phylogenetic Bray–Curtis distance metrics for the overall functional gene profiles between THE CONT and TUL-treated piglets. The percent variation explained by each principal component is indicated on the axes.

FIGURE S5 Heatmap of identified antimicrobial resistance genes in the fecal microbiota at class level of each piglet in both control (CONT) and tulathromycin (TUL) groups. The yellow/blue color of the X axis of the heat map represent the degree of similarities and cluster between the related class of assessed parameters.

FIGURE S6 The difference in bacterial diversity indices (Chao 1, Shannon, Simpson and Pielou’s evenness indices) measures between the control (CONT) and tulathromycin (TUL) piglets at different sampling days (0, 5, and 20). The individual data points, which represent bacterial diversity for each piglet, are depicted. Error bars represent the standard errors.

TABLE S1 The most predominant 100 microbial species across all the samples in both control (CONT) and tulathromycin (TUL) treated piglets.

## References

[B1] Allen H. K. Trachsel J. Looft T. Casey T. A. (2014). Finding alternatives to antibiotics. *Ann. N. Y. Acad. Sci.* 1323 91–100. 10.1111/nyas.12468 24953233

[B2] Andrews S. (2010). *Babraham Bioinformatics - FastQC A Quality Control Tool for High Throughput Sequence Data.* Available at: http://www.bioinformatics.babraham.ac.uk/projects/fastqc/ (accessed November 9 2018).

[B3] Benchaoui H. A. Nowakowski M. Sherington J. Rowan T. G. Sunderland S. J. (2004). Pharmacokinetics and lung tissue concentrations of tulathromycin in swine. *J. Vet. Pharmacol. Ther.* 27 203–210. 10.1111/j.1365-2885.2004.00586.x 15305848

[B4] Bolger A. M. Lohse M. Usadel B. (2014). Trimmomatic: a flexible trimmer for illumina sequence data. *Bioinformatics* 30 2114–2120. 10.1093/bioinformatics/btu170 24695404 PMC4103590

[B5] Chambers L. Yang Y. Littier H. Ray P. Zhang T. Pruden A. (2015). Metagenomic analysis of antibiotic resistance genes in dairy cow feces following therapeutic administration of third generation cephalosporin. *PLoS One* 10:e0133764. 10.1371/journal.pone.0133764 26258869 PMC4530880

[B6] Choo J. M. Abell G. C. J. Thomson R. Morgan L. Waterer G. Gordon D. L. (2018). Impact of long-term erythromycin therapy on the oropharyngeal microbiome and resistance gene reservoir in non-cystic fibrosis bronchiectasis. *mSphere* 3 e103–e118. 10.1128/mSphere.00103-18 29669883 PMC5907653

[B7] Clausen P. T. L. C. Zankari E. Aarestrup F. M. Lund O. (2016). Benchmarking of methods for identification of antimicrobial resistance genes in bacterial whole genome data. *J. Antimicrob. Chemother.* 71 2484–2488. 10.1093/jac/dkw184 27365186

[B8] Connelly S. Subramanian P. Hasan N. A. Colwell R. R. Kaleko M. (2018). Distinct consequences of amoxicillin and ertapenem exposure in the porcine gut microbiome. *Anaerobe* 53 82–93. 10.1016/j.anaerobe.2018.04.012 29689301

[B9] Cromwell G. L. (2002). Why and how antibiotics are used in swine production. *Anim. Biotechnol.* 13 7–27. 10.1081/abio-120005767 12212945

[B10] Czaplewski L. Bax R. Clokie M. Dawson M. Fairhead H. Fischetti V. A. (2016). Alternatives to antibiotics-a pipeline portfolio review. *Lancet Infect. Dis.* 16 239–251. 10.1016/S1473-3099(15)00466-1 26795692

[B11] Dhariwal A. Chong J. Habib S. King I. L. Agellon L. B. Xia J. (2017). MicrobiomeAnalyst: a web-based tool for comprehensive statistical, visual and meta-analysis of microbiome data. *Nucleic Acids Res.* 45 W180–W188. 10.1093/nar/gkx295 28449106 PMC5570177

[B12] Doster E. Rovira P. Noyes N. R. Burgess B. A. Yang X. Weinroth M. D. (2018). Investigating effects of tulathromycin metaphylaxis on the fecal resistome and microbiome of commercial feedlot cattle early in the feeding period. *Front. Microbiol.* 9:1715. 10.3389/fmicb.2018.01715 30105011 PMC6077226

[B13] Ferguson J. F. Liu Y. Reilly M. P. Xue C. Gregory B. Li M. (2013). Evaluating the impact of sequencing depth on transcriptome profiling in human adipose. *PLoS One* 8:e66883. 10.1371/journal.pone.0066883 23826166 PMC3691247

[B14] Founou L. L. Founou R. C. Essack S. Y. (2016). Antibiotic resistance in the food chain: a developing country-perspective. *Front. Microbiol.* 7:1881. 10.3389/fmicb.2016.01881 27933044 PMC5120092

[B15] Gaeta N. C. Lima S. F. Teixeira A. G. Ganda E. K. Oikonomou G. Gregory L. (2017). Deciphering upper respiratory tract microbiota complexity in healthy calves and calves that develop respiratory disease using shotgun metagenomics. *J. Dairy Sci.* 100 1445–1458. 10.3168/jds.2016-11522 27988122

[B16] Glass E. M. Wilkening J. Wilke A. Antonopoulos D. Meyer F. (2010). Using the metagenomics RAST server (MG-RAST) for analyzing shotgun metagenomes. *Cold Spring Harb. Protoc.* 5 1–11. 10.1101/pdb.prot5368 20150127

[B17] Hoelzer K. Wong N. Thomas J. Talkington K. Jungman E. Coukell A. (2017). Antimicrobial drug use in food-producing animals and associated human health risks: what, and how strong, is the evidence? *BMC Vet. Res.* 13:211. 10.1186/s12917-017-1131-1133 28676125 PMC5496648

[B18] Inouye M. Dashnow H. Raven L.-A. Schultz M. B. Pope B. J. Tomita T. (2014). SRST2: rapid genomic surveillance for public health and hospital microbiology labs Michael. *Genome Med.* 6:90. 10.1111/1469-0691.12217 25422674 PMC4237778

[B19] Kelly J. Daly K. Moran A. W. Ryan S. Bravo D. Shirazi-Beechey S. P. (2017). Composition and diversity of mucosa-associated microbiota along the entire length of the pig gastrointestinal tract; dietary influences. *Environ. Microbiol.* 19 1425–1438. 10.1111/1462-2920.13619 27871148

[B20] Kubasova T. Davidova-Gerzova L. Merlot E. Medvecky M. Polansky O. Gardan-Salmon D. (2017). Housing systems influence gut microbiota composition of sows but not of their piglets. *PLoS One* 12:e0170051. 10.1371/journal.pone.0170051 28085934 PMC5234784

[B21] Lopez-Rojas R. Kempf M. Rolain J.-M. Diene S. M. Landraud L. Gupta S. K. (2013). ARG-ANNOT, a new bioinformatic tool to discover antibiotic resistance genes in bacterial genomes. *Antimicrob. Agents Chemother.* 58 212–220. 10.1128/AAC.01310-13 24145532 PMC3910750

[B22] MacKie R. I. Koike S. Krapac I. Chee-Sanford J. Maxwell S. Aminov R. I. (2006). Tetracycline residues and tetracycline resistance genes in groundwater impacted by swine production facilities. *Anim. Biotechnol.* 17 157–176. 10.1080/10495390600956953 17127527

[B23] Maradiaga N. Aldridge B. Zeineldin M. Lowe J. (2018). Gastrointestinal microbiota and mucosal immune gene expression in neonatal pigs reared in a cross-fostering model. *Microb. Pathog.* 121 27–39. 10.1016/j.micpath.2018.05.007 29742464

[B24] Maron D. F. Smith T. J. S. Nachman K. E. (2013). Restrictions on antimicrobial use in food animal production: an international regulatory and economic survey. *Global. Health* 9:48. 10.1186/1744-8603-9-48 24131666 PMC3853314

[B25] Mazzei T. Mini E. Noveffi A. Periti P. (1993). Chemistry and mode of action of macrolides SS ˆ ST Clanthrcnycin. *J. Antimicrob. Chemother.* 31(Suppl. C) 1–9. 10.1093/jac/31.suppl_C.1 7683018

[B26] McEwen S. A. Fedorka-Cray P. J. (2002). Antimicrobial use and resistance in animals. *Clin Infect Dis* 34(Suppl. 3) S93–S106. 10.1086/340246 11988879

[B27] Pantoja-Feliciano I. G. Clemente J. C. Costello E. K. Perez M. E. Blaser M. J. Knight R. (2013). Biphasic assembly of the murine intestinal microbiota during early development. *ISME J.* 7 1112–1115. 10.1038/ismej.2013.15 23535917 PMC3660675

[B28] Parks D. H. Tyson G. W. Hugenholtz P. Beiko R. G. (2014). STAMP: statistical analysis of taxonomic and functional profiles. *Bioinformatics* 30 3123–3124. 10.1093/bioinformatics/btu494 25061070 PMC4609014

[B29] Pérez-Cobas A. E. Artacho A. Knecht H. Ferrús M. L. Friedrichs A. Ott S. J. (2013). Differential effects of antibiotic therapy on the structure and function of human gut microbiota. *PLoS One* 8:e80201. 10.1371/journal.pone.0080201 24282523 PMC3839934

[B30] Phillips I. Casewell M. Cox T. De Groot B. Friis C. Jones R. (2004). Does the use of antibiotics in food animals pose a risk to human health? A critical review of published data. *J. Antimicrob. Chemother.* 53 28–52. 10.1093/jac/dkg483 14657094

[B31] Pyörälä S. Baptiste K. E. Catry B. van Duijkeren E. Greko C. Moreno M. A. (2014). Macrolides and lincosamides in cattle and pigs: use and development of antimicrobial resistance. *Vet. J.* 200 230–239. 10.1016/j.tvjl.2014.02.028 24685099

[B32] Qin J. Li R. Raes J. Arumugam M. Burgdorf K. S. Manichanh C. (2010). A human gut microbial gene catalogue established by metagenomic sequencing. *Nature* 464 59–65. 10.1038/nature08821 20203603 PMC3779803

[B33] Rodríguez J. M. Murphy K. Stanton C. Ross R. P. Kober O. I. Juge N. (2015). The composition of the gut microbiota throughout life, with an emphasis on early life. *Microb. Ecol. Heal. Dis.* 26 1–17. 10.3402/mehd.v26.26050 25651996 PMC4315782

[B34] Schokker D. Zhang J. Zhang L. L. Vastenhouw S. A. Heilig H. G. Smidt H. (2014). Early-life environmental variation affects intestinal microbiota and immune development in new-born piglets. *PLoS One* 9:e100040. 10.1371/journal.pone.0100040 24941112 PMC4062469

[B35] Segata N. Izard J. Waldron L. Gevers D. Miropolsky L. Garrett W. S. (2011). Metagenomic biomarker discovery and explanation. *Genome Biol.* 12:R60. 10.1186/gb-2011-12-6-r60 21702898 PMC3218848

[B36] Slifierz M. J. Friendship R. M. Weese J. S. (2015). Longitudinal study of the early-life fecal and nasal microbiotas of the domestic pig. *BMC Microbiol.* 15:184. 10.1186/s12866-015-0512-517 26391877 PMC4578254

[B37] Thanner S. Drissner D. Walsh F. (2016). Antimicrobial resistance in agriculture. *MBio* 7 e02227-15. 10.1128/mBio.02227-15 27094336 PMC4850276

[B38] Thomas M. Webb M. Ghimire S. Blair A. Olson K. Fenske G. J. (2017). Metagenomic characterization of the effect of feed additives on the gut microbiome and antibiotic resistome of feedlot cattle. *Sci. Rep.* 7 1–13. 10.1038/s41598-017-12481-6 28947833 PMC5612972

[B39] Tsukayama P. Boolchandani M. Patel S. Pehrsson E. C. Gibson M. K. Chiou K. L. (2018). Characterization of wild and captive baboon gut microbiota and their antibiotic resistomes. *mSystems* 3 e00016–e00018. 10.1128/mSystems.00016-18 29963641 PMC6020475

[B40] Yassour M. Vatanen T. Siljander H. Hamalainen A.-M. Harkonen T. Ryhanen S. J. (2016). Natural history of the infant gut microbiome and impact of antibiotic treatment on bacterial strain diversity and stability. *Sci. Transl. Med.* 8:343ra81. 10.1126/scitranslmed.aad0917 27306663 PMC5032909

[B41] Zeineldin M. Aldridge B. Blair B. Kancer K. Lowe J. (2018). Impact of parenteral antimicrobial administration on the structure and diversity of the fecal microbiota of growing pigs. *Microb. Pathog.* 118 220–229. 10.1016/j.micpath.2018.03.035 29578067

[B42] Zeineldin M. Lowe J. de Godoy M. Maradiaga N. Ramirez C. Ghanem M. (2017a). Disparity in the nasopharyngeal microbiota between healthy cattle on feed, at entry processing and with respiratory disease. *Vet. Microbiol.* 208 30–37. 10.1016/j.vetmic.2017.07.006 28888646

[B43] Zeineldin M. M. Lowe J. F. Grimmer E. D. de Godoy M. R. C. Ghanem M. M. Abd El-Raof Y. M. (2017b). Relationship between nasopharyngeal and bronchoalveolar microbial communities in clinically healthy feedlot cattle. *BMC Microbiol.* 17:138. 10.1186/s12866-017-1042-2 28645257 PMC5481913

[B44] Zhao Y. Su J.-Q. An X.-L. Huang F.-Y. Rensing C. Brandt K. K. (2017). Feed additives shift gut microbiota and enrich antibiotic resistance in swine gut. *Sci. Total Environ.* 621 1224–1232. 10.1016/j.scitotenv.2017.10.106 29054657

