# Supplementary figures and images for: Negligible Impact of Perinatal Tulathromycin Metaphylaxis on the Developmental Dynamics of Fecal Microbiota and Their Accompanying Antimicrobial Resistome in Piglets
Source: Front Microbiol. 2019 Apr 5;10:726. doi: 10.3389/fmicb.2019.00726 (PMC6460945; doi:10.3389/fmicb.2019.00726)

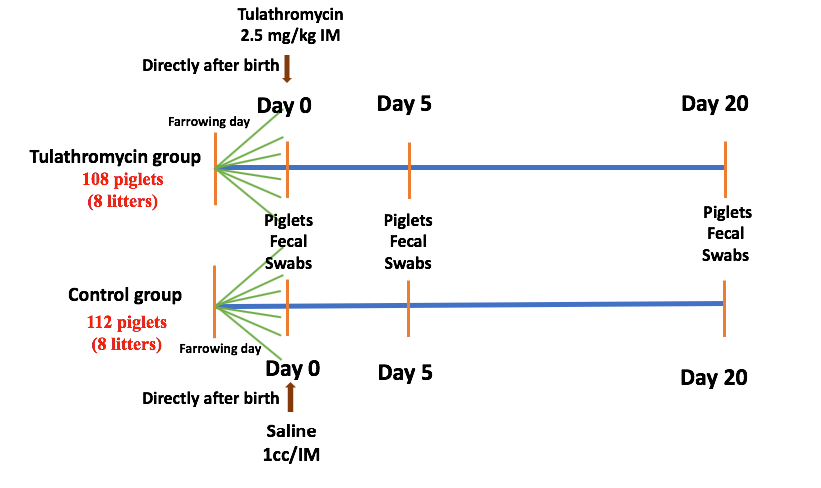

Supplement: FIGURE S1 — The detailed experimental design. [file Image_1.TIF]

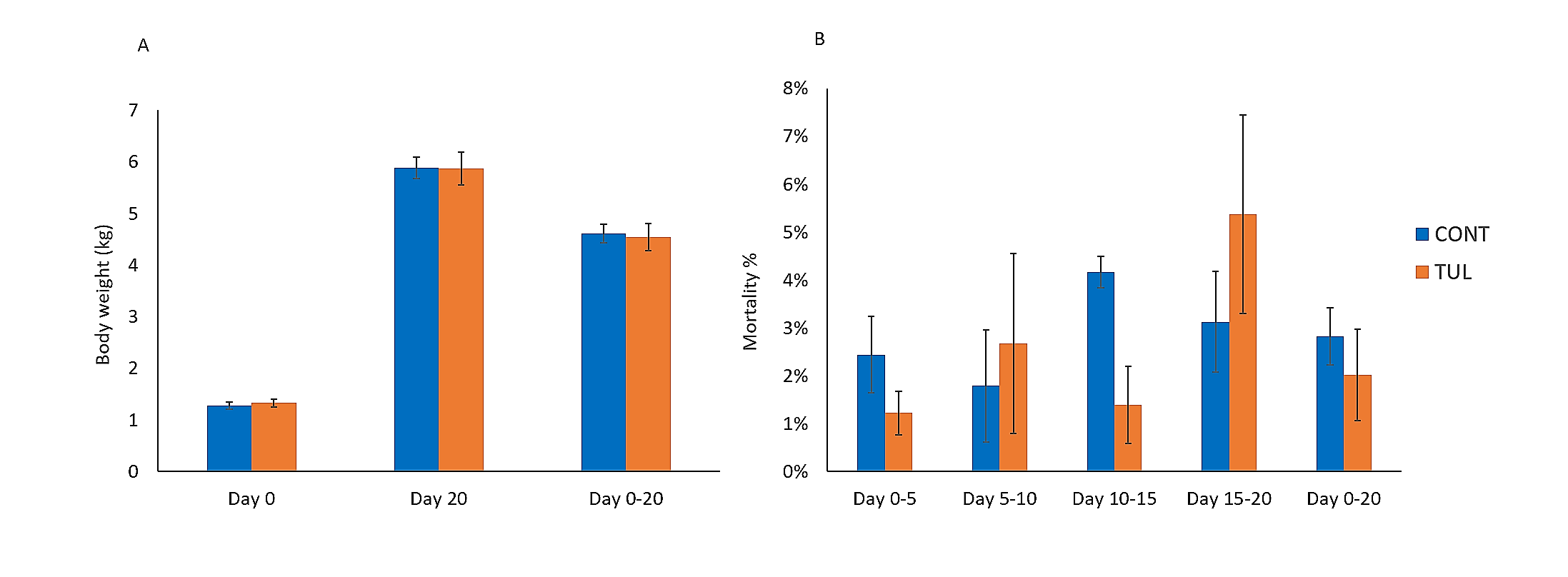

Supplement: FIGURE S2 — (A) Bar graph illustrating the body weight (kg) at day 0 and day 20, and the average weight gain from day 0 to day 20 of age. (B) Bar graph illustrating the Mortality percent of piglets from day 0 to day 5 (day 0–5), from day 5 to day 10 (day 5–10), from day 10 to day 15 (day 10–15), and from day 15 to day 20 (day 15–20). The piglets were treated with a single dose of TUL at day 0 soon after birth (TUL, N = 108), or treated with saline (CONT, N = 113). There was no significant change in the average daily weight gain and overall mortality ratio (P > 0.05). [file Image_2.TIF]

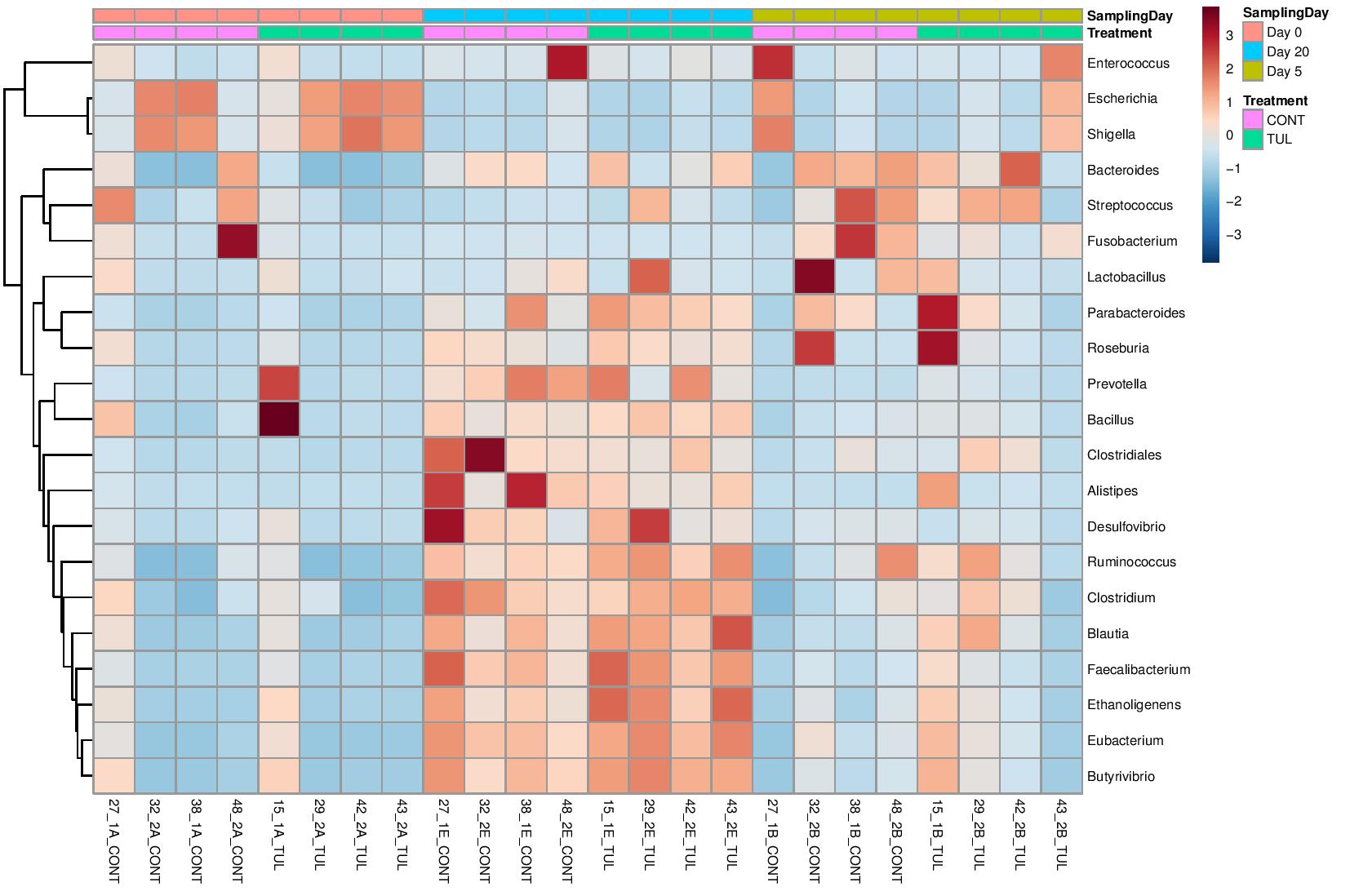

Supplement: FIGURE S3 — Taxonomic classification of shotgun metagenomic sequences at the genus level for the control (CONT) and tulathromycin (TUL) treated piglets at each sampling time days (0, 5, and 20). Only those bacterial genera that averaged more than 1% of the relative abundance across all samples are displayed. [file Image_3.JPEG]

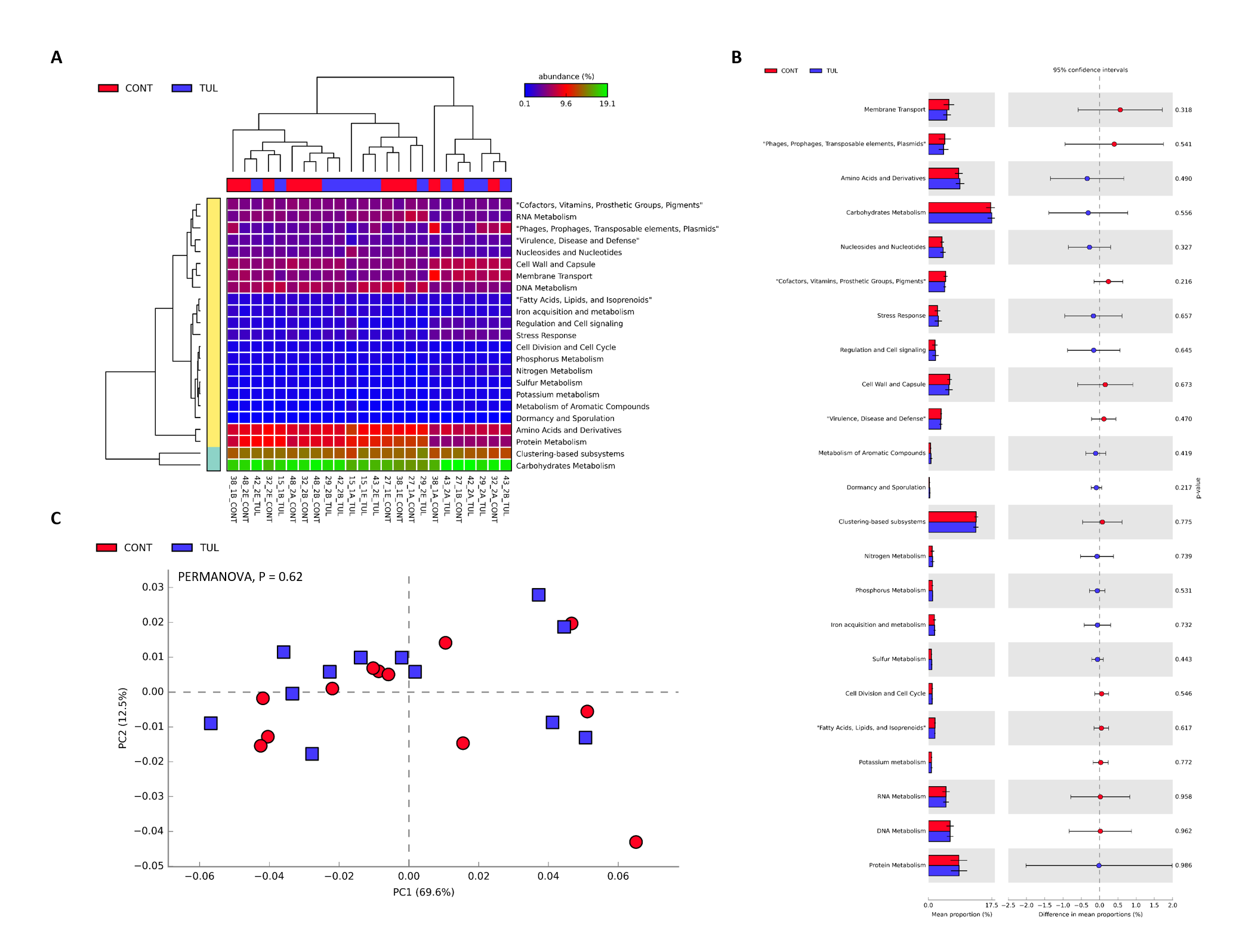

Supplement: FIGURE S4 — Inferred predictive functional features of piglet’s fecal microbiota. (A) Heatmap cluster analysis of metagenomic functional capability at level 2 KEGG pathway based on differentially abundant functional features between the control (CONT) and tulathromycin (TUL) groups, and at different sampling days (0, 5, and 20). The yellow/blue color of the X axis of the heat map represent the degree of similarities and cluster between the related class of assessed parameters. (B) The relative abundance of the functional profiles at level 2 in the TUL treated piglets compared to the CONT group (P-value > 0.05). (C) Principal component analysis (PCA) based on non-phylogenetic Bray–Curtis distance metrics for the overall functional gene profiles between THE CONT and TUL-treated piglets. The percent variation explained by each principal component is indicated on the axes. [file Image_4.TIF]

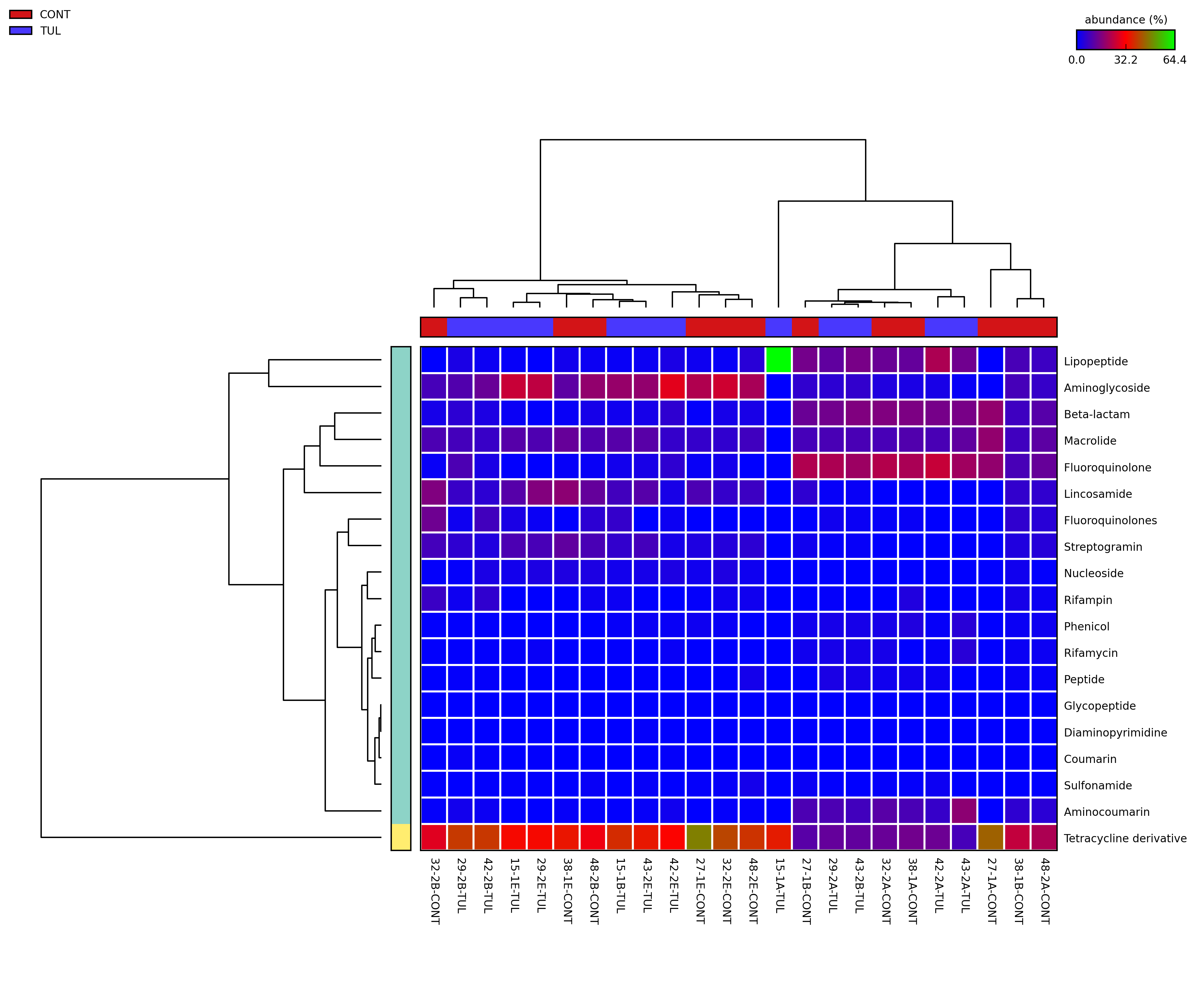

Supplement: FIGURE S5 — Heatmap of identified antimicrobial resistance genes in the fecal microbiota at class level of each piglet in both control (CONT) and tulathromycin (TUL) groups. The yellow/blue color of the X axis of the heat map represent the degree of similarities and cluster between the related class of assessed parameters. [file Image_5.PNG]

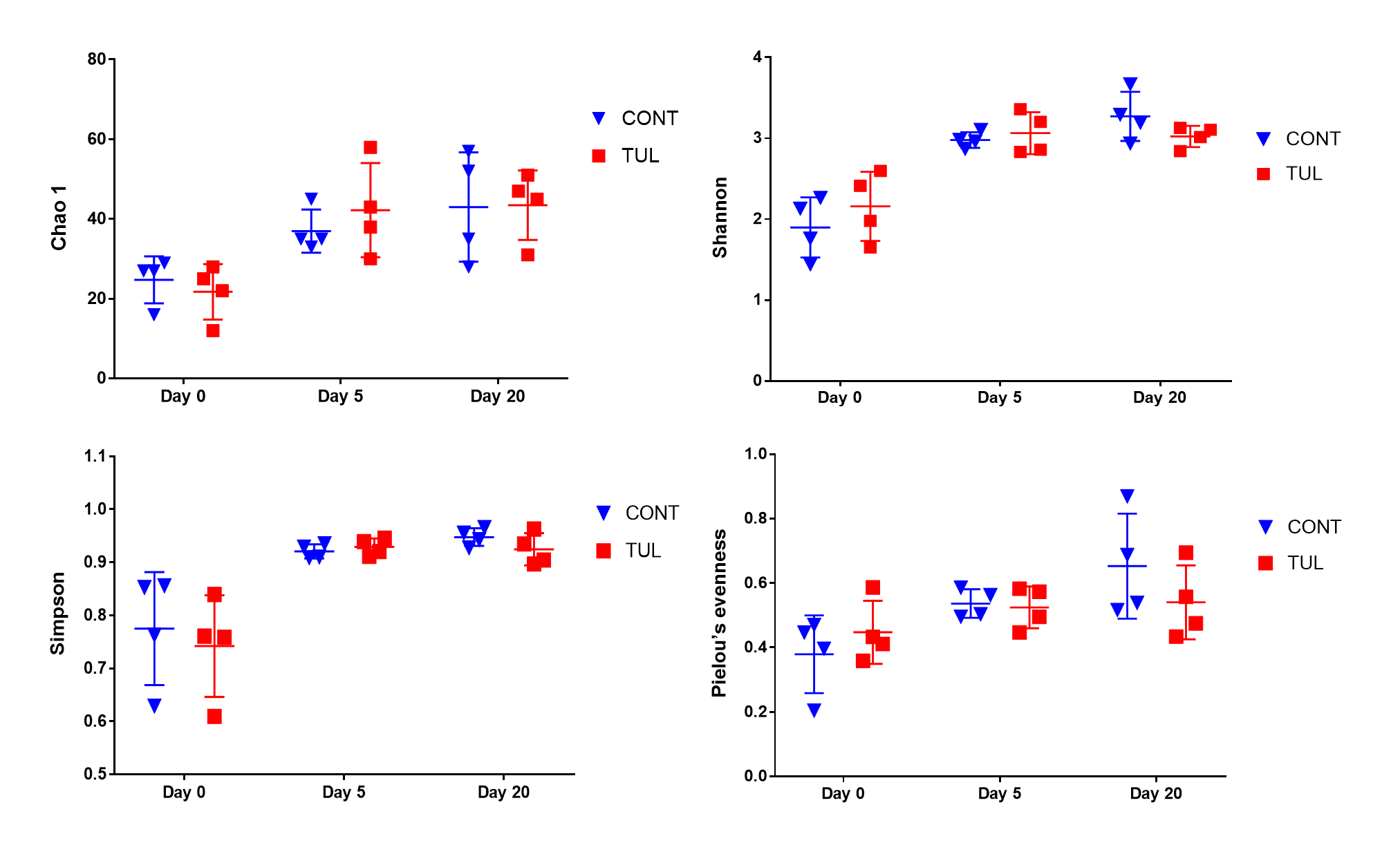

Supplement: FIGURE S6 — The difference in bacterial diversity indices (Chao 1, Shannon, Simpson and Pielou’s evenness indices) measures between the control (CONT) and tulathromycin (TUL) piglets at different sampling days (0, 5, and 20). The individual data points, which represent bacterial diversity for each piglet, are depicted. Error bars represent the standard errors. [file Image_6.TIF]
