# Supplementary material for: Negligible Impact of Perinatal Tulathromycin Metaphylaxis on the Developmental Dynamics of Fecal Microbiota and Their Accompanying Antimicrobial Resistome in Piglets
Source: Front Microbiol. 2019 Apr 5;10:726. doi: 10.3389/fmicb.2019.00726 (PMC6460945; doi:10.3389/fmicb.2019.00726)
Supplement: TABLE S1 — The most predominant 100 microbial species across all the samples in both control (CONT) and tulathromycin (TUL) treated piglets. [file Table_1.docx]

**Table S1.** The most predominant 100 microbial species across all the samples in both CONT and TUL treated piglets.

| **Species level** | **CONT-0** | **CONT-5** | **CONT-20** | **TUL-0** | **TUL-5** | **TUL- 20** |
| --- | --- | --- | --- | --- | --- | --- |
| *Escherichia coli* | 41.47% | 18.08% | 4.60% | 44.46% | 16.71% | 2.60% |
| *Bacteroides fragilis* | 4.41% | 6.22% | 3.04% | 0.55% | 8.06% | 4.42% |
| *Clostridium saccharolyticum* | 0.27% | 1.21% | 3.86% | 0.09% | 1.91% | 2.61% |
| *Bacteroides vulgatus* | 0.43% | 3.17% | 0.91% | 0.19% | 2.77% | 0.88% |
| *Clostridium perfringens* | 3.00% | 1.32% | 0.42% | 2.86% | 1.33% | 0.59% |
| *Bacteroides sp. 2_1_16* | 2.12% | 1.17% | 0.99% | 0.12% | 1.80% | 1.56% |
| *Clostridium bolteae* | 0.24% | 1.21% | 3.31% | 0.09% | 1.80% | 1.65% |
| *Clostridium scindens* | 0.13% | 0.95% | 2.18% | 0.07% | 1.17% | 1.84% |
| *Clostridium hathewayi* | 0.13% | 0.43% | 2.05% | 0.05% | 0.93% | 3.08% |
| *Bacteroides sp. 1_1_6* | 0.34% | 1.60% | 1.00% | 0.12% | 1.51% | 1.09% |
| *Bacteroides thetaiotaomicron* | 0.31% | 1.59% | 0.93% | 0.08% | 1.47% | 0.90% |
| *Streptococcus gallolyticus* | 0.70% | 1.57% | 0.37% | 0.18% | 1.23% | 0.79% |
| *Clostridiales bacterium 1_47FAA* | 0.10% | 0.63% | 2.54% | 0.04% | 1.07% | 1.15% |
| *Lactobacillus reuteri* | 0.12% | 1.38% | 0.60% | 0.07% | 0.54% | 1.34% |
| *Bacteroides sp. 4_3_47FAA* | 0.22% | 1.69% | 0.53% | 0.10% | 1.43% | 0.47% |
| *Clostridium difficile* | 0.78% | 0.62% | 0.93% | 0.33% | 1.17% | 1.05% |
| *Eubacterium rectale* | 0.13% | 0.74% | 1.14% | 0.07% | 0.97% | 1.34% |
| *Clostridium botulinum* | 0.43% | 0.68% | 0.72% | 0.38% | 1.11% | 0.95% |
| *Lactobacillus delbrueckii* | 0.06% | 2.73% | 0.11% | 0.04% | 0.38% | 0.14% |
| *Bacteroides uniformis* | 0.14% | 2.03% | 0.46% | 0.06% | 0.67% | 0.34% |
| *Clostridium phytofermentans* | 0.12% | 0.53% | 1.23% | 0.07% | 0.80% | 1.31% |
| *Bacteroides capillosus* | 0.08% | 0.42% | 1.33% | 0.08% | 0.59% | 1.55% |
| *Parabacteroides distasonis* | 0.09% | 0.92% | 0.81% | 0.08% | 0.79% | 0.95% |
| *Fusobacterium nucleatum* | 0.99% | 1.18% | 0.22% | 0.03% | 0.40% | 0.18% |
| *Bacteroides sp. 3_2_5* | 1.10% | 0.39% | 0.45% | 0.06% | 0.78% | 0.70% |
| *Fusobacterium varium* | 0.49% | 1.62% | 0.05% | 0.03% | 0.49% | 0.03% |
| *Shigella flexneri* | 1.99% | 1.05% | 0.23% | 2.38% | 0.85% | 0.13% |
| *Ruminococcus gnavus* | 0.41% | 0.59% | 0.40% | 0.05% | 0.88% | 0.53% |
| *Roseburia inulinivorans* | 0.08% | 0.85% | 0.39% | 0.04% | 0.90% | 0.58% |
| *Parabacteroides merdae* | 0.06% | 0.28% | 0.38% | 0.03% | 1.16% | 0.83% |
| *Fusobacterium mortiferum* | 1.07% | 0.65% | 0.02% | 0.03% | 0.47% | 0.04% |
| *Subdoligranulum variabile* | 0.03% | 0.11% | 0.57% | 0.03% | 0.17% | 1.91% |
| *Ruminococcaceae bacterium D16* | 0.06% | 0.37% | 0.95% | 0.05% | 0.44% | 0.93% |
| *Neisseria polysaccharea* | 10.33% | 1.77% | 0.98% | 17.37% | 0.69% | 0.29% |
| *Bacteroides sp. D20* | 0.09% | 1.28% | 0.29% | 0.06% | 0.44% | 0.22% |
| *Clostridium leptum* | 0.04% | 0.23% | 0.49% | 0.04% | 0.38% | 1.28% |
| *Lactobacillus vaginalis* | 0.05% | 0.16% | 0.46% | 0.02% | 0.31% | 1.02% |
| *Enterococcus faecalis* | 0.16% | 2.03% | 1.60% | 0.09% | 1.09% | 0.49% |
| *Ruminococcus albus* | 0.06% | 0.22% | 0.68% | 0.05% | 0.34% | 1.03% |
| *Salmonella enterica* | 1.29% | 0.66% | 0.22% | 1.64% | 0.72% | 0.16% |
| *Clostridium beijerinckii* | 0.24% | 0.34% | 0.47% | 0.28% | 0.52% | 0.61% |
| *Faecalibacterium prausnitzii* | 0.05% | 0.21% | 0.72% | 0.04% | 0.33% | 0.95% |
| *Escherichia sp. 3_2_53FAA* | 1.39% | 0.67% | 0.15% | 1.60% | 0.62% | 0.12% |
| *Bacteroides dorei* | 0.10% | 0.66% | 0.36% | 0.07% | 0.60% | 0.32% |
| *Eubacterium limosum* | 0.08% | 0.20% | 0.56% | 0.04% | 0.30% | 0.92% |
| *Clostridium asparagiforme* | 0.07% | 0.27% | 0.83% | 0.03% | 0.40% | 0.68% |
| *Clostridium thermocellum* | 0.07% | 0.23% | 0.57% | 0.07% | 0.30% | 0.84% |
| *Ethanoligenens harbinense* | 0.05% | 0.21% | 0.54% | 0.05% | 0.36% | 0.87% |
| *Parabacteroides sp. D13* | 0.05% | 0.51% | 0.44% | 0.01% | 0.39% | 0.51% |
| *Bacteroides sp. 20_3* | 0.03% | 0.40% | 0.50% | 0.03% | 0.41% | 0.57% |
| *Alistipes putredinis* | 0.02% | 0.02% | 1.09% | 0.01% | 0.38% | 0.56% |
| *Streptococcus infantarius* | 0.25% | 0.58% | 0.02% | 0.11% | 0.58% | 0.05% |
| *Holdemania filiformis* | 0.06% | 0.19% | 0.62% | 0.03% | 0.32% | 0.71% |
| *Ruminococcus torques* | 0.04% | 0.68% | 0.19% | 0.03% | 0.44% | 0.31% |
| *Desulfitobacterium hafniense* | 0.09% | 0.21% | 0.56% | 0.06% | 0.31% | 0.64% |
| *Clostridium proteoclasticum* | 0.07% | 0.21% | 0.52% | 0.06% | 0.34% | 0.63% |
| *Streptococcus suis* | 0.39% | 0.42% | 0.10% | 0.19% | 0.58% | 0.11% |
| *Ilyobacter polytropus* | 0.40% | 0.62% | 0.06% | 0.02% | 0.18% | 0.07% |
| *Shigella dysenteriae* | 1.08% | 0.51% | 0.13% | 1.16% | 0.45% | 0.07% |
| *Eubacterium eligens* | 0.06% | 0.24% | 0.50% | 0.03% | 0.34% | 0.57% |
| *Clostridium nexile* | 0.08% | 0.26% | 0.37% | 0.02% | 0.45% | 0.50% |
| *Bacteroides ovatus* | 0.19% | 0.30% | 0.36% | 0.02% | 0.37% | 0.28% |
| *Anaerotruncus colihominis* | 0.04% | 0.13% | 0.73% | 0.01% | 0.26% | 0.56% |
| *Clostridium sp. M62/1* | 0.09% | 0.21% | 0.43% | 0.02% | 0.34% | 0.44% |
| *Clostridium kluyveri* | 0.08% | 0.22% | 0.35% | 0.09% | 0.33% | 0.46% |
| *Marvinbryantia formatexigens* | 0.02% | 0.15% | 0.44% | 0.03% | 0.26% | 0.58% |
| *Shigella sonnei* | 0.89% | 0.46% | 0.10% | 1.11% | 0.38% | 0.06% |
| *Bacteroides plebeius* | 0.06% | 0.41% | 0.27% | 0.02% | 0.32% | 0.28% |
| *Prevotella ruminicola* | 0.03% | 0.09% | 0.69% | 0.13% | 0.17% | 0.61% |
| *Enterococcus faecium* | 0.17% | 0.24% | 0.32% | 0.08% | 0.69% | 0.29% |
| *Streptococcus pyogenes* | 0.37% | 0.30% | 0.19% | 0.05% | 0.30% | 0.29% |
| *Lactobacillus acidophilus* | 0.06% | 0.87% | 0.05% | 0.09% | 0.09% | 0.07% |
| *Parabacteroides johnsonii* | 0.04% | 0.10% | 0.26% | 0.03% | 0.32% | 0.54% |
| *Pyramidobacter piscolens* | 0.02% | 0.02% | 0.72% | 0.01% | 0.02% | 0.62% |
| *Bacteroides sp. 2_2_4* | 0.13% | 0.32% | 0.23% | 0.02% | 0.30% | 0.19% |
| *Bacteroides stercoris* | 0.03% | 0.30% | 0.26% | 0.01% | 0.32% | 0.32% |
| *Prevotella melaninogenica* | 0.03% | 0.07% | 0.63% | 0.07% | 0.16% | 0.60% |
| *Clostridium sp. 7_2_43FAA* | 0.11% | 0.12% | 0.18% | 0.26% | 0.24% | 0.41% |
| *Bacteroides sp. 2_1_7* | 0.02% | 0.34% | 0.24% | 0.03% | 0.24% | 0.30% |
| *Roseburia intestinalis* | 0.05% | 0.16% | 0.37% | 0.01% | 0.25% | 0.39% |
| *Clostridium cellulolyticum* | 0.05% | 0.13% | 0.34% | 0.04% | 0.18% | 0.46% |
| *Alkaliphilus metalliredigens* | 0.06% | 0.16% | 0.31% | 0.05% | 0.19% | 0.38% |
| *Fusobacterium ulcerans* | 0.26% | 0.48% | 0.04% | 0.06% | 0.10% | 0.04% |
| *Streptococcus agalactiae* | 0.18% | 0.31% | 0.09% | 0.07% | 0.27% | 0.15% |
| *Porphyromonas gingivalis* | 0.02% | 0.06% | 0.50% | 0.10% | 0.20% | 0.40% |
| *Klebsiella pneumoniae* | 0.31% | 0.81% | 0.05% | 0.34% | 0.22% | 0.71% |
| *Bacteroides intestinalis* | 0.05% | 0.25% | 0.28% | 0.02% | 0.27% | 0.24% |
| *Lactobacillus johnsonii* | 0.02% | 0.17% | 0.08% | 0.03% | 0.62% | 0.18% |
| *Lactobacillus crispatus* | 0.02% | 0.70% | 0.04% | 0.03% | 0.05% | 0.05% |
| *Streptococcus mutans* | 0.14% | 0.28% | 0.08% | 0.04% | 0.25% | 0.14% |
| *Lactobacillus amylovorus* | 0.08% | 0.69% | 0.02% | 0.11% | 0.06% | 0.04% |
| *Blautia hansenii* | 0.06% | 0.10% | 0.23% | 0.03% | 0.29% | 0.37% |
| *Clostridium methylpentosum* | 0.02% | 0.13% | 0.24% | 0.01% | 0.23% | 0.38% |
| *Bacteroides coprocola* | 0.03% | 0.10% | 0.28% | 0.03% | 0.43% | 0.24% |
| *Clostridium acetobutylicum* | 0.07% | 0.15% | 0.21% | 0.07% | 0.23% | 0.29% |
| *Desulfovibrio desulfuricans* | 0.03% | 0.10% | 0.34% | 0.03% | 0.08% | 0.38% |
| *Streptococcus equinus* | 0.27% | 0.29% | 0.07% | 0.02% | 0.12% | 0.11% |
| *Clostridium hylemonae* | 0.02% | 0.12% | 0.29% | 0.01% | 0.21% | 0.35% |
| *Blautia hydrogenotrophica* | 0.01% | 0.10% | 0.28% | 0.03% | 0.17% | 0.38% |
| *Bacteroides cellulosilyticus* | 0.04% | 0.21% | 0.26% | 0.01% | 0.23% | 0.23% |
